# Reply to: differential diagnosis of pseudohypopyon and discussion of extranodal natural killer/T-cell lymphoma presenting as hypopyon panuveitis

**DOI:** 10.1186/s12886-022-02616-3

**Published:** 2022-10-04

**Authors:** Nutchaya Sukon, Nattaporn Tesavibul, Pitipol Choopong, Noppakhun Panyayingyong, Sutasinee Boonsopon

**Affiliations:** 1 Department of Ophthalmology, Faculty of Medicine, Siriraj Hospital, Mahidol University, 2 Wanglang Road, Bangkoknoi, Bangkok, 10700 Thailand; 2 Metta Pracharak Hospital (Wat Rai Khing), 52, Moo 2, Rai Khing sub-district, Sampran District, Nakhonpathom, 73210 Thailand

## Abstract

Extranodal natural killer/T-cell lymphoma rarely presents as intraocular masquerade syndrome. We thank Dr. Evereklioglu for bringing up the importance of a thorough ocular examination, differential diagnosis, and consideration of the characteristics of ocular masquerade syndrome.

## Main text

We thank Dr. Evereklioglu for his comments regarding our case report of an intraocular extranodal natural killer/T-cell lymphoma (ENKTL) [1], and we are happy to address his main points here. First, Dr. Evereklioglu questioned the terminology of “hypopyon” that we used in the article. We agree that careful examination to distinguish between true hypopyon and pseudohypopyon would be beneficial. In our defense, we believed most ophthalmologists would understand that our patient did not have true hypopyon, since we clearly explained that the definite diagnosis in our patient was ENKTL. Indeed, in our case report, we aimed to demonstrate an atypical presentation and the disease progression of intraocular ENKTL. We did not wish to mislead ophthalmologists with the terminology we used. Also, many articles published worldwide have used the term hypopyon to describe the findings in ocular masquerade syndrome [2–5], and we believed the readers would understand the distinction.

Next, Dr. Evereklioglu brought up a point about “uninjected white eye”, and he proposed that this should be used to exclude an infectious etiology of ocular inflammation. We think this is partially correct, and we are not opposing that subtle conjunctival injection is a clue to diagnose ocular masquerade syndrome, but we also raise the point that some patients with infectious uveitis may also present with mild conjunctival injection [6–8]. In addition, we did not mention the prior treatment of our patient in the article. She was receiving an hourly topical prednisolone acetate, which may partially have reduced ocular surface inflammation at presentation. Lastly, Dr. Evereklioglu commented on the differential diagnosis that we made, specifically endogenous endophthalmitis (EE), and criticized that EE should not be included in our differential diagnosis. In our defense, the symptoms of EE vary from mild discomfort to severe eye pain and severe visual loss [9, 10]. In one systematic review, it was reported that up to 33% of patients with EE were initially misdiagnosed as noninfectious uveitis, conjunctivitis, and others [11]. Unlike exogenous endophthalmitis, painless ocular inflammation may not always be used to exclude EE and therefore we included EE in our differential diagnosis.

## Data Availability

The dataset used during the current study is available from the corresponding author upon reasonable request.

## Abbreviations

EE: Endogenous endophthalmitis
ENKTL: Extranodal natural killer/T-cell lymphoma
